# CT characteristics and clinical implications of acute type A aortic intramural hematoma

**DOI:** 10.3389/fcvm.2022.1041796

**Published:** 2023-01-09

**Authors:** Hsu-Ting Yen, Chia-Chen Wu, Yi-Wei Lee, Chien-Ming Lo, Yen-Yu Chen

**Affiliations:** ^1^Division of Thoracic and Cardiovascular Surgery, Department of Surgery, Kaohsiung Chang Gung Memorial Hospital and Chang Gung University College of Medicine, Kaohsiung, Taiwan; ^2^Department of Radiology, Kaohsiung Chang Gung Memorial Hospital and Chang Gung University College of Medicine, Kaohsiung, Taiwan

**Keywords:** type A aortic intramural hematoma, computarized tomography, ulcer-like projection, acute aortic syndrome (AAS), penetrating atherosclerotic ulcer, sudden death

## Abstract

**Objectives:**

Computed tomography (CT) has been increasingly used in the diagnosis of acute aortic syndrome, and a number of high-risk CT imaging features have been reported. We aimed to identify CT imaging findings suggesting high-risk for acute aortic syndrome by examining clinical outcomes of patients with acute type A aortic intramural hematoma (TAIMH).

**Methods:**

This retrospective study analyzed the relationship of clinical patient characteristics and imaging features with mortality and aortic events in 63 patients receiving initial medical treatment for TAIMH. Multivariate regression analysis was used to determine the predictors of aortic events, and the Kaplan–Meier method was used to analyze survival and aortic events.

**Results:**

During a median follow-up of 4.2 years, 25 patients experienced aortic events and 40% of these occurred within 7 days of admission. In total, 12 patients experienced aortic death and 12 patients underwent open aortic surgery or endovascular stenting for aortic disease. In multivariate regression analysis, penetrating atherosclerotic ulcers (PAUs) or ulcer-like projections (ULPs) (*P* = 0.04) and pericardial effusion (*P* = 0.03) were independent predictors of aortic events. In the Cox regression model, PAUs/ULPs (*P* = 0.04) and pericardial effusion (*P* = 0.04) were independently associated with lower aortic event-free survival.

**Conclusion:**

Identification of high-risk CT features is important for clinical decision-making during TAIMH treatment. Early and frequent CT imaging follow-up is required in patients receiving medical treatment. PAUs/ULP and pericardial effusion were the strongest predictors of adverse aortic events.

## 1. Introduction

Acute type A aortic intramural hematoma (TAIMH) presents with clinical features that are distinct from those of the classic acute type A aortic dissection (TAAD) (1). The incidence of TAIMH in Eastern Asian, which accounts for 22–44% of all type A acute aortic syndrome cases, is significantly higher than that in Western countries (2). Although the underlying cause remains unclear, clinical outcomes of TAIMH are better than those of TAAD, which may be attributable to lower rates of aortic regurgitation, coronary involvement, and malperfusion syndrome. However, pericardial effusion and cardiac tamponade are more frequent in TAIMH than in TAAD (3, 4). Based on a high in-hospital mortality of 40–50% in patients with TAIMH who receive medical treatment, urgent open surgery within the first 24 h after diagnosis is the class I recommendation for most patients (3, 5–7). However, some studies, predominantly from South Korea and Japan, showed favorable results with medical therapy in TAIMH (1, 4, 8, 9). Initial medical therapy with a “watch-and-wait” strategy may be a reasonable option in patients without complications, particularly in elderly patients without high-risk radiologic features (6, 10–12).

Multidetector-row computed tomography (CT), which is increasingly used in the diagnosis of TAIMH, provides more detailed information for clinical decision-making in TAIMH treatment. Some imaging characteristics, such as penetrating atherosclerotic ulcers (PAUs) and ulcer-like projections (ULPs) (13, 14), enlarged aortic diameter, and increased hematoma thickness are commonly reported features associated with adverse events (8, 15–17). However, these studies are limited to relatively small number of patients (<50), and clinical studies including Chinese patients with TAIMH are lacking.

In the present study, we retrospectively evaluated the clinical outcomes of 63 patients with TAIMH receiving initial medical treatment to investigate CT imaging features associated with poor prognosis.

## 2. Materials and methods

### 2.1. Patients and study design

This retrospective cohort study was approved by the Institutional Review Board of Chang Gung Medical Foundation (approval no: 202000716B0). Informed consent was waived because of the retrospective nature of the study and analyses of anonymous clinical data. Between January 1, 2004 and December 31, 2019, 391 consecutive patients with Stanford type A acute aortic syndrome were admitted within 2 weeks of symptomatic onset. Of these, 35 patients without available contrast-enhanced CT scans were excluded. Patients who met at least one of the following criteria were included: (i) presence of intramural hematoma (IMH), defined as crescentic or circular wall thickening (≥5 mm) in the ascending aorta on precontrast CT images, which was not enhanced during the arterial or delayed phase (*n* = 81) and (ii) presence of thrombosed retrograde type A aortic dissection (RTAD), defined as completely or partially thrombosed false lumen of the ascending aorta with dissection originating distal to the ascending aorta on contrast-enhanced CT images (*n* = 34). Patients fulfilling the following criteria were excluded: (i) emergent open aortic surgery (*n* = 34) or endovascular stenting (*n* = 7); (ii) initial presentation with cardiac arrest, aortic rupture, myocardial infarction, acute aortic regurgitation, or major end-organ ischemia, such as that of the brain or intestine (*n* = 8); and (iii) signed do-not-resuscitate consent form and refusal of further management (*n* = 3). Patients presenting without pain or organ ischemia, in whom CT scan findings suggested aortitis based on the absence of aortic wall hyperdensity on precontrast phase, were excluded. Patients treated with open surgical drainage or percutaneous pericardiocentesis for cardiac tamponade were included. Emergent surgery was defined as that performed within 8 h following diagnosis.

### 2.2. Initial CT scans protocol and follow-up

In all but one patient, initial CT scans were performed within 2 days after the initial onset of chest or back pain or the emergence of organ hypoperfusion. In 20 patients, CT scans were performed in referring hospitals. In our institution, a variety of multidetector-row CT scanners were used over the years and included 4-detector row CT scanners (Somatom Volume Zoom, Siemens Healthineers, Germany; Lightspeed, GE Healthcare, USA), 64-detector row CT scanners (Somatom Definition AS, Siemens Healthineers, Germany; Aquilion 64, Toshiba Medical Systems, Japan), and dual-source CT scanner Somatom Definition Flash scanners, Siemens Healthineers, Germany). The scan range extended from the thoracic inlet to kidneys or the femoral head. Electrocardiographic gating was not used. Each scan included unenhanced and contrast-enhanced acquisitions using bolus tracking technique. Non-ionic contrast medium (350 mgI/ml) was administered with intravenous bolus injection (70–100 ml) at a rate of 2–4 ml/s. Arterial-phase scans started after the threshold was reached 150 HU at the descending aorta, and venous-phase scans started 90 s after the arterial phase scans. The following scanning parameters were used: tube voltage, 100–130 kVp; tube current, 150–280 mAs with dose modulation; pitch, 0.6–1.2; rotation time, 0.5 s; and slice reconstruction, 2–5 mm. Coronal and sagittal oblique views were routinely reconstructed for assessment after 2009. Forty patients received repeat CT imaging with or without contrast enhancement within 7 days of admission. In total, 47 patients underwent follow-up CT imaging before discharge and 6 patients underwent follow-up CT imaging after discharge at our institution. The remaining ten patients did not undergo follow-up CT imaging in our institution.

### 2.3. Definitions and data collection

TAAD was defined as a characteristic contrast-enhanced double-channel aorta with an intimal flap involving the ascending aorta. PAUs were characterized as crater-like or mushroom-shaped focal protrusions of the irregular intima with atherosclerotic plaques (18). Small PAUs were defined as crater-like ulcerations with a depth of <3 mm. ULPs were defined as localized blood-filled pouches protruding into the thrombosed false lumen with a wide communicating orifice of >3 mm and an adjacent wall lacking atherosclerotic changes (18). Maximal hematoma thickness (MHT), defined as the maximal thickness of the hematoma on axial CT image of the ascending aorta, was measured from the intima to the adventitia. Maximal aortic diameter (MAD) was measured on the same slice as the MHT and was defined as the maximum wall-to-wall diameter of the ascending aorta, which was usually located close to the bifurcation of the left and right pulmonary arteries. Baseline demographic and clinical characteristics of patients, including age, sex, body mass index, and comorbidities, were collected from the medical records.

The primary endpoint was mortality and the secondary endpoint was aortic event. Aortic events included aortic rupture, evolution to TAAD, requirement of open aortic surgery or endovascular stenting for progressing aortic disease, and aortic death. Aortic death was defined as death from an aortic event or unexplained sudden death. Early aortic events were those occurring within the admission period, and late aortic events were those occurring after discharge. The follow-up period ended in December 31, 2020 or at the time of death or aortic events. In all enrolled patients, 1-year follow-up data were obtained through clinical follow-up or telephone contact with the patients or their relatives.

### 2.4. Statistical analysis

Categorical variables were expressed as numbers with percentages and compared using the chi-squared or Fisher’s exact test. Continuous variables were expressed as means ± standard deviation and compared using Student’s *t* or the Mann–Whitney *U* test. Univariate analyses were performed to identify factors predicting aortic events. Variables with a statistically significant difference at a *P* value of <0.10 were included in multivariate stepwise logistic regression analysis, and results were expressed as odds ratios (ORs) with 95% confidence intervals (CIs). For continuous variables identified as predictors of aortic events, receiver operating characteristic curves were generated and area under the receiver operating characteristic curve (AUC) and the highest Youden’s index were calculated to determine cutoff values. Overall survival was defined as the time from the date of diagnosis to the date of death or last follow-up. Aortic event-free survival was defined as the time from the date of diagnosis to the date of aortic event. The Kaplan–Meier method was used for survival analysis of mortality and aortic events, and between-group differences were examined using the log-rank test. The Cox proportional hazards regression analysis was used to determine hazard ratios (HRs) of independent predictors of aortic event-free survival. All analyses were conducted using IBM SPSS (version 25.0 for Windows; SPSS, Chicago, IL, USA), and statistical significance was set at a *P* value of <0.05.

## 3. Results

### 3.1. Patient characteristics and outcomes

The study cohort was comprised of 63 patients, including 53 patients with IMH and 10 patients with RTAD. The mean age was 63.5 (range, 33–89) years, and 34 patients (54%) were male. Most patients (86%) had a history of hypertension. The mean MHT and MAD were 9.3 ± 4.2 and 46.3 ± 6.1 mm, respectively. Among these patients, cardiac tamponade was reported in three patients and open pericardiostomy and percutaneous pericardiocentesis were performed in two and one patient, respectively. Comparison between the patients with and without aortic events is summarized in Table 1. During the median follow-up of 4.2 (range, 0–15.7) years, 25 patients (40%) experienced aortic events. During the admission period, 13 patients (21%) experienced early aortic events, including 6 patients who experienced sudden death and 7 patients who underwent open aortic surgery or endovascular stenting for progressing aortic disease. Of the six patients with sudden death, five patients died within 5 days of admission and one patient experienced sudden unexplained death on hospital day 12 without progression of aortic disease or cardiac tamponade; therefore, follow-up CT scans were not obtained from these six patients. Six patients underwent open aortic surgery, including three patients with the indication of hematoma progression or relapse, two patients with evolution to TAAD, and one patient with delayed cardiac tamponade. One patient underwent endovascular stenting for newly developed ULP in distal descending aorta identified with follow-up CT scan.

**TABLE 1:** Baseline demographics and clinical outcomes of patients.

Characteristics	All (*n* = 63)	Aortic events		*P* value
			
		No (*n* = 38)	Yes (*n* = 25)	
Age (years), mean (SD)	63.5 (11.5)	62.2 (10.0)	65.5 (13.5)	0.302
Male, *n* (%)	34 (54.0)	22 (57.9)	12 (48.0)	0.441
BMI (kg/m^2^), mean (SD)	26.4 (3.9)	26.2 (4.1)	26.7 (3.6)	0.650
Smoking, *n* (%)	20 (31.7)	12 (31.6)	8 (32.0)	0.972
Hypertension, *n* (%)	54 (85.7)	30 (78.9)	24 (96.0)	0.075
Diabetes mellitus, *n* (%)	10 (15.9)	7 (18.4)	3 (12.0)	0.727
Chronic kidney disease, *n* (%)	15 (23.8)	9 (23.7)	6 (24.0)	0.977
Liver cirrhosis, *n* (%)	1 (1.6)	1 (2.6)	0 (0.0)	1.000
Cerebrovascular disease, *n* (%)	8 (12.7)	6 (15.8)	2 (8.0)	0.461
Congestive heart failure, *n* (%)	2 (3.2)	2 (5.3)	0 (0.0)	0.514
Initial CT findings				
RTAD type, *n* (%)	9 (14.3)	5 (13.2)	4 (16.0)	1.000
Presence of PAU/ULP^a^, *n* (%)	25 (39.7)	11 (28.9)	14 (56.0)	0.032
In the zone 0–1, *n* (%)	10 (15.9)	4 (10.5)	6 (24.0)	0.176
Beyond zone 2, *n* (%)	18 (28.6)	9 (23.7)	9 (36.0)	0.290
Periaortic hematoma, *n* (%)	4 (6.3)	2 (5.3)	2 (8.0)	1.000
Pericardial effusion, *n* (%)	23 (36.5)	7 (18.4)	16 (64.0)	<0.001
Cardiac tamponade, *n* (%)	3 (4.8)	1 (2.6)	2 (8.0)	0.557
Pleural effusion, *n* (%)	8 (12.7)	5 (13.2)	3 (12.0)	1.000
Descending aorta involvement, *n* (%)	51 (81.0)	35 (92.1)	16 (64.0)	0.008
MHT (mm), mean (SD)	9.3 (4.2)	9.2 (4.1)	9.4 (4.4)	0.960
MAD (mm), mean (SD)	46.3 (6.1)	44.8 (4.8)	48.4 (7.3)	0.045
Hospital stay (days), mean (SD)	16.8 (14.0)	16.2 (12.2)	17.8 (16.6)	0.966
In-hospital death, *n* (%)	9 (14.3)	3 (7.9)	6 (24.0)	0.138

BMI, body mass index; CT, computerized tomography; MAD, maximal aortic diameter; MHT, maximal hematoma thickness; PAU, penetrating atherosclerotic ulcer; RTAD, thrombosed retrograde type A aortic dissection; SD, standard deviation; ULP, ulcer-like projection.

^a^Two patients had multiple thoracic aorta PAUs and one patient had concomitant ascending ULP and distal arch PAU.

In patients requiring aortic surgery for early aortic events, the mean duration between hospital admission and surgery was 8.3 (range, 1–25) days. Before discharge, three patients died due to non-aortic causes, including one patient with moderate/severe chronic kidney disease who died due to pneumonia-related sepsis on hospital day 18; one patient who died due to major stroke, sepsis, and acute kidney injury on hospital day 36; and one patient who required renal replacement therapy and ventilator support and died on hospital day 72. Two patients underwent elective open aortic surgery for a dilated ascending aorta of >4 cm and one patient underwent endovascular stenting for residual type B aortic dissection during the admission period. There was no mortality among the patients who underwent delayed or elective surgery. The in-hospital and aortic event-related mortality rates were 14.3 and 9.5%, respectively.

Of the surviving patients, 12 (22%) developed late aortic events at a median of 7 (range 2–90) months after diagnosis. Five patients underwent thoracic endovascular aortic or open repair for enlarged pseudoaneurysm or dissecting aneurysm at a median of 5 (range, 2–8) months after diagnosis. In six patients, late aortic death occurred at a median follow-up of 38.5 (range, 4–90) months after diagnosis and was due to pseudoaneurysm rupture or newly developed TAAD in four patients, descending aortic aneurysm rupture in one patient, and static focal dissection in the ascending aorta concomitant with a descending aortic aneurysm of >56 mm in one patient who experienced sudden death at home. One patient with distal ascending ULP who developed TAAD was alive at 105-month follow-up evaluation.

**FIGURE 1:**
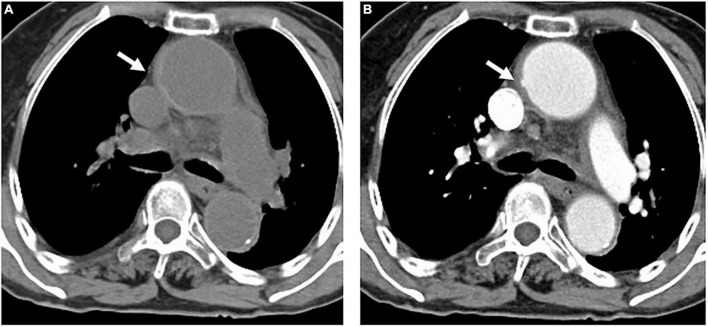
An 84-year-old woman with TAIMH and tiny ULP. A precontrast CT scan showed circular high attenuation aortic wall thickening (5 mm) (arrow) involving the ascending aorta (49 mm) **(A)**. A tiny ULP (arrow) was observed in the greater curvature of the ascending aorta **(B)**. Follow-up CT was not performed and the patient suffered from sudden death on the fourth day of admission. CT, computerized tomography; TAIMH, type A aortic intramural hematoma; ULP, ulcer-like projection.

**FIGURE 2:**
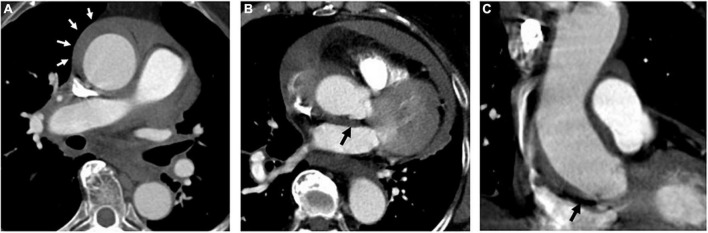
A 74-year-old woman with TAIMH and unnoticeable ULP. A contrast CT scan showed an ascending aorta aneurysm (52 mm) with a circular aortic wall thickening (9 mm) (white arrow) **(A)**. Contrast-enhanced CT scan in the axial **(B)** and coronal view **(C)** showed an ULP (black arrow) at the ascending aorta just above the aortic root. The patient suffered from sudden death on the first day of admission. CT, computerized tomography; TAIMH, type A aortic intramural hematoma; ULP, ulcer-like projection.

**FIGURE 3:**
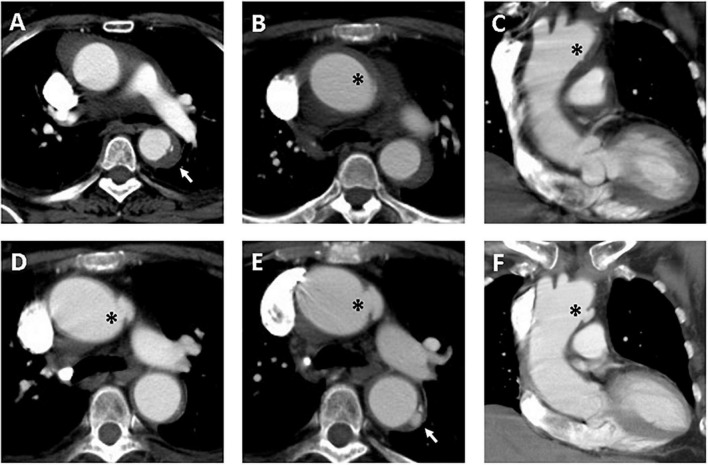
Serial CT scans showed progression of PAU and ULP in a 69-year-old woman with TAIMH. Contrast-enhanced CT scan showed TAIMH with PAU (arrow) in the proximal descending aorta **(A)** and ULP (asterisk) in the proximal aortic arch **(B,C)**. Three months later, the follow-up CT scan showed enlarged ULP (**D**, asterisk). The PAU (arrow) in the proximal descending aorta and ULP (asterisk) in the proximal aortic arch were slowly progressed in overall size on 14-month follow-up CT scan **(E,F)**. The patient received medical treatment alone and was still alive.

**FIGURE 4:**
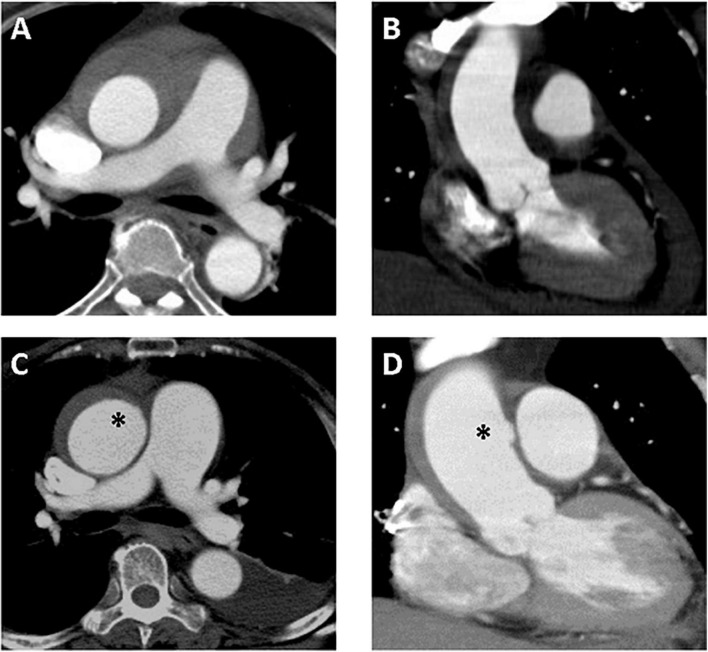
A 61-year-old woman with TAIMH and newly developed ULP. Type A intramural hematoma (thickness 12 mm) was identified on initial contrast-enhanced CT scan and no focal intimal disruption was visible in the axial **(A)** and coronal view **(B)**. Follow-up CT scan obtained on sixth day of admission showed an irregular margin of aortic wall (asterisk) in the axial view **(C)** that a newly developed ULP (asterisk) was confirmed in the coronal view **(D)**. The patient suffered from sudden death on the 12th day of admission. CT, computerized tomography; TAIMH, type A aortic intramural hematoma; ULP, ulcer-like projection.

In the entire cohort, PAUs or ULPs in the ascending aorta or the proximal aortic arch were observed on initial CT scan in ten patients (16%; Figures 1–3) and on follow-up CT scan in four patients (6%; Figure 4). Aortic deaths occurred in 7 of these 14 patients (50%), including 5 in-hospital deaths and 2 late deaths. The rates of PAUs/ULPs, pleural effusion, and absence of descending aorta involvement were significantly higher in patients with aortic events than in those without aortic events (*P* = 0.03, *P* < 0.001, and *P* = 0.01, respectively); the MAD was also significantly larger in patients with aortic events than in those without aortic events (*P* = 0.045).

### 3.2. Predictors of aortic events and survival

The multivariate stepwise logistic regression analysis performed with adjustment for age and sex revealed that PAUs/ULPs (OR 4.200, 95% CI 1.040–16.961, *P* = 0.04) and pericardial effusion (OR 5.107, 95% CI 1.204–21.667, *P* = 0.03) were independent predictors of aortic events (Table 2).

**TABLE 2:** Multivariate logistic regression analysis for predicting aortic events.

	Univariate analysis		Multivariate analysis	
		
Variables	OR (95% CI)	*P* value	OR (95% CI)	*P* value
Age (year)	1.026 (0.980–1.074)	0.269	0.961 (0.898–1.027)	0.237
Male	0.671 (0.243–1.853)	0.442	1.053 (0.284–3.897)	0.939
BMI (kg/m^2^)	1.032 (0.904–1.178)	0.644		
RTAD type	1.257 (0.303–5.222)	0.753		
Presence of PAU/ULP	3.124 (1.087–8.981)	0.034	4.200 (1.040–16.961)	0.044
PAU/ULP in zone 0–1	2.684 (0.673–10.713)	0.162		
Periaortic hematoma	1.565 (0.206–11.901)	0.665		
Pericardial effusion	7.873 (2.474–25.050)	<0.001	5.107 (1.204–21.667)	0.027
Cardiac tamponade	3.217 (0.276–37.517)	0.351		
Pleural effusion	0.900 (0.195–4.155)	0.893		
Absent DesAo involvement	6.562 (1.564–27.540)	0.010	4.588 (0.755–27.894)	0.098
MHT (mm)	1.009 (0.894–1.139)	0.879		
MAD (mm)	1.116 (1.009–1.235)	0.033	1.072 (0.920–1.250)	0.370

AsAo, ascending aorta; BMI, body mass index; CI, confidence interval; MAD, maximal aortic diameter; MHT, maximal hematoma thickness; OR, odds ratio; PAU, penetrating atherosclerotic ulcer; RTAD, thrombosed retrograde type A aortic dissection; ULP, ulcer-like projection.

**FIGURE 5:**
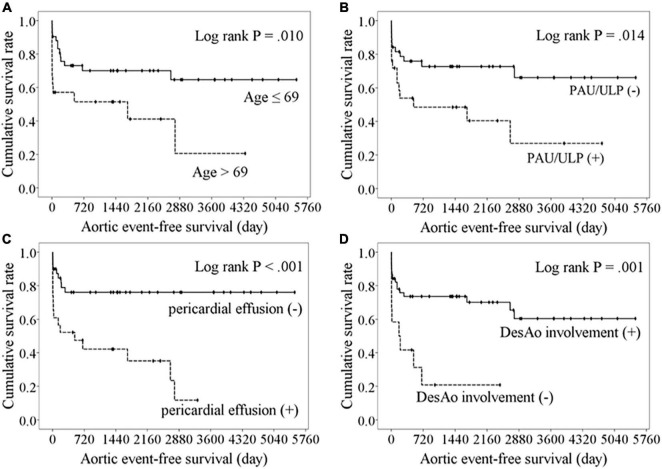
Kaplan–Meier analysis of freedom from aortic events comparing with the variables. Lower aortic event-free survival were observed in patients with aged over 69 years **(A)**, the presence of PAU or ULP **(B)**, pericardial effusion **(C)**, or absence of descending aorta involvement **(D)**.

The cumulative overall survival rates were 79.4, 67.7, and 60.3% at 1, 5, and 10 years, respectively, and the aortic event-free survival rates were 67.4, 60.7, and 52.3% at 1, 5, and 10 years, respectively. The optimal cutoff values for age and MAD to predict aortic events were 69 years (AUC 0.579, sensitivity 48.0%, specificity 76.3%, *P* = 0.29) and 46 mm (AUC 0.649, sensitivity 64.0%, specificity 57.9%, *P* = 0.04), respectively. The Kaplan–Meier analysis comparing aortic event-free survival between the groups (Figure 5) revealed age >69 years (*P* = 0.01, log-rank test), PAUs/ULPs (*P* = 0.01, log-rank test), pericardial effusion (*P* < 0.001, log-rank test), and absence of descending aorta involvement (*P* = 0.001, log-rank test) as poor prognostic factors. The Cox regression analysis indicated that PAUs/ULPs (HR 2.417, 95% CI 1.063–5.494, *P* = 0.04) and pericardial effusion (HR 2.678, 95% CI 1.044–6.870, *P* = 0.04) were independent predictors of lower aortic event-free survival.

## 4. Discussion

The present retrospective cohort study, which included a relatively large number of Chinese patients who initially received medical treatment for TAIMH, reveals several important findings. First, adverse events were relatively common (40%) and included some that were fatal. Second, PAUs/ULPs and pericardial effusion were the significant independent predictors of early and late aortic events and affected aortic event-free survival independent of the MHT and MAD. Third, multidetector-row CT scans could be used in identifying high-risk imaging features and in clinical decision-making. However, sudden death may occur even in the absence of these high-risk radiologic features on the initial CT scan. Thereafter, close follow-up with imaging studies is mandatory.

Over the last two decades, real-world observational studies on IMH have provided valid and useful information on TAIMH management; however, optimal initial treatment strategies remain controversial. The most important concern is the potential risk of sudden death. Song et al. reported that 5 of 85 consecutive medically treated patients with TAIMH (5.9%) experienced sudden death during the acute stage (1). Hata et al. reported that 13.6% of 66 patients with TAIMH receiving initial medical treatment experienced sudden death due to cardiac tamponade (19). In the present study, six patients (9.5%) experienced sudden death during admission and one patient (1.6%) with delayed cardiac tamponade was rescued by emergent surgery. In the present study, the most probable causes of sudden death are aortic rupture and cardiac tamponade, although other causes cannot be ruled out. Risk factors of sudden death might be influenced by differences in the inclusion criteria employed among the previous studies. A relatively lower risk of sudden death (4–6%) was observed in studies by Song et al. (1) and Kitai et al. (8) which excluded patients with PAUs in the ascending aorta or the aortic arch. Initial MAD of ≥55 mm and MHT of ≥16 mm were predictors of adverse events in the observational study by Song et al. (1). In the present study, all six patients experiencing sudden death had pericardial effusion and five of the six patients had PAUs or ULPs in the ascending aorta. Only one of these patients had a dilated aortic diameter of ≥55 mm, whereas two patients exhibited a larger MHT of ≥16 mm. Therefore, PAUs/ULPs and pericardial effusion were convincing predictors of aortic events in the present study.

Advances in imaging technologies led to an increase in the identification of intimal tears on CT scans in patients with IMH. Nevertheless, some microintimal tears might not be easily detected on the initial CT scan. In a retrospective review of 106 patients undergoing open aortic surgery for TAIMH, 56 patients presented with ULPs in the ascending aorta or the aortic arch on preoperative CT scans, and 1 patient presented with PAU in the aortic arch. During surgery, 81 patients had intimal tears in the ascending aorta or the aortic arch; 24 of these 81 patients did not have ULPs or PAUs suggesting intimal tears on preoperative CT scans (20). In the present study, new ULPs in the ascending aorta were detected in four patients, including one patient who experienced sudden death during admission and two patients who experienced aortic death at 6 and 22 months of follow-up, respectively. Kitamura et al. considered that the “watch-and-wait” strategy with frequent CT scans and timely aortic intervention were important to improve outcomes (10).

The difference between PAUs and ULPs has been rarely investigated. PAU is defined as an ulcerating atherosclerotic lesion that penetrates the intima to reach the media and may be surrounded by localized hematoma. PAUs, which are sometimes multifocal, are uncommon in the ascending aorta. Unlike PAUs, ULPs suggests small intimal tears without atherosclerosis that might be present on initial or follow-up CT scans. Differentiating PAUs associated with IMH from ULPs with a thrombosed false lumen may be difficult on the initial CT scan. Several studies showed that PAUs and ULPs, particularly those located in the ascending aorta or the aortic arch, were associated with poor prognosis (21, 22). In a study of 36 patients with TAIMH, including 11 patients with ULPs in the ascending aorta on initial or follow-up CT scans, Lee et al. reported that 7 patients experienced adverse events, including the need for emergency surgery in 2 patients and the development of TAAD in 5 patients (22). Additionally, they reported that localized aortic dissection in the distal ascending aorta could remain stable on long-term follow-up in some cases (22). Our results agree with those of previous studies. The present study cohort included a relatively large number of patients with PAUs (*n* = 1) or ULPs (*n* = 13) in the ascending aorta or the aortic arch; of these, seven patients (50%) died due to aortic disease whereas four patients (28.6%) remained alive with chronic TAAD or focal dissection in the ascending aorta during a mean follow-up of 2.8 years. Although several experts suggest that PAUs and ULPs have distinct pathogenic mechanisms and clinical course, the limited data in the current study cannot shed further light in that aspect.

To date, several predictors have been repeatedly reported to be associated with adverse aortic events, such as an MAD of >45–55 mm (1, 8, 16, 17) and an MHT of >8–16 mm (1, 9, 15, 17). In the present study, the rate of descending aorta involvement was lower and the MAD was larger in patients with aortic events. These imaging characteristics might reflect higher false lumen pressure and thinner aortic wall, which appear to predispose patients to blood leakage outside the adventitia, subsequently resulting in pericardial effusion. In the present study, the patients with pericardial effusion were significantly older, had larger MADs, and had less frequency involvement of the descending aorta, suggesting that patients experiencing pericardial effusion and aortic events shared similar characteristics and imaging findings. The prognostic role of MAD and MHT remains uncertain; however, pericardial effusion was shown to be an important independent predictor of aortic events (23). In that study including 172 patients from China, the authors also showed that pericardial effusion was an independent predictor of adverse aortic events. Moreover, another study utilized several variables, including ULP, systolic blood pressure, ascending aorta diameter, and pericardial effusion, to establish a novel risk score to predict cardiovascular death and surgery in patients with TAIMH (24).

In the present cohort, most fatal adverse events occurred within the hyperacute phase of TAIMH and most patients exhibited high-risk radiologic features on initial CT scan. Multidetector-row CT scan is useful for the detection of microintimal defects in patients with TAIMH. Early and frequent CT imaging follow-up is reasonable for patients without complications receiving initial medical treatment. Emergent open aortic repair should be recommended in patients with PAUs or ULPs and pericardial effusion on CT scan, which have emerged as significant predictors of adverse aortic events in the present study.

This study has several limitations. First, this was an observational study conducted in a single center and our results may not apply to other populations, especially European and North American cohorts. Second, this was a retrospective study, which might have led to a selection bias. We excluded patients who underwent emergent surgery, which might have affected clinical outcomes. Third, there were no standard medical care strategies, CT examination protocols, and follow-up planning. The current study results should only be considered for the generation of hypotheses for future studies. Fourth, there might be some biases regarding CT characteristics based on the limitations of imaging technologies during the study period. Although these data suggest that PAUs/ULPs and pericardial effusion on initial CT scan were the strongest predictors of adverse aortic events, optimal patient management was not clarified in the present study.

In conclusion, TAIMH accounted for a large proportion of patients with type A acute aortic syndrome. Adverse aortic events were common in patients receiving initial medical treatment, and most of the fatal adverse events occurred within the acute phase. Multidetector-row CT scan was useful in the diagnosis of TAIMH and could provide valid information for clinical decision-making in TAIMH treatment. PAUs/ULPs on initial or follow-up CT scan, especially in the ascending aorta or aortic arch, and pericardial effusion were the strongest predictors of adverse aortic events. Early and frequent follow-up with CT scans is important in identifying these high-risk imaging features.

## Data availability statement

The original contributions presented in this study are included in the article/supplementary material, further inquiries can be directed to the corresponding author.

## Ethics statement

The studies involving human participants were reviewed and approved by Institutional Review Board of Chang Gung Medical Foundation (202000716B0). Written informed consent for participation was not required for this study in accordance with the national legislation and the institutional requirements.

## Author contributions

Y-YC: conceptualization, formal analysis, writing—review and editing, and supervision. H-TY: writing—original draft preparation and project administration. C-CW and C-ML: validation, data curation, and resources. Y-WL: methodology, software, investigation, and visualization. All authors contributed to the article and approved the submitted version.
